# The challenges of interrogating adipose tissue extracellular vesicle functions in physiology

**DOI:** 10.1038/s42003-022-03511-9

**Published:** 2022-06-14

**Authors:** Clair Crewe

**Affiliations:** 1 Department of Cell Biology and Physiology, Washington University School of Medicine, St. Louis, MO USA; 2 Department of Internal Medicine, Division of Endocrinology, Metabolism and Lipid Research, Washington University School of Medicine, St. Louis, MO USA

**Keywords:** Metabolism, Cellular imaging

## Abstract

Recent developments in adipose tissue-derived extracellular vesicle (EV) research is highlighted, assessing current adipose tissue EV research strategies and obstacles the field faces.

## Teaser

Adipose tissue-derived extracellular vesicles (EVs) are at the center of the budding field of EV signaling in metabolic regulation for their strong regulatory effects on local and systemic metabolism. This essay will highlight seminal studies in adipose tissue EV research, critically assess current adipose tissue EV research strategies and discuss the obstacles the field faces moving forward.

Adipose tissue (AT) EVs are sourced by directly isolating EVs from adipose tissue or from harvesting culture media from incubating AT explants. This highly heterogeneous population of EVs derives mainly from adipocytes^1^ but also endothelial cells, fibroblasts, macrophages and other immune cells. Like all EVs, AT EVs are phospholipid-enclosed vesicles loaded with bioactive molecules spanning miRNAs, mRNAs, DNA, proteins, lipids, and metabolites. As such, these EVs can have potent signaling effects on receiving cells. Pioneering studies demonstrated that EVs isolated from human and mouse AT explants and immortalized adipocytes induced inflammation and insulin resistance in myocytes and hepatocytes, with more pronounced effects if the AT came from obese subjects^2–5^. This data has held up over time as a recent study has shown that both human plasma- and subcutaneous AT-derived small EVs (sEVs) from obese humans with nonalcoholic fatty liver disease induce insulin resistance in primary myotubes and hepatocytes^6^. Although early studies did explore the effect of AT EVs on systemic metabolism by injecting purified EVs into mice^2^, it was not until the study by Thomou et. al. that a significant role for circulating adipocyte-derived EVs was established in vivo^7^. This study used a mouse model where dicer was knocked out specifically in adipocytes to suppress miRNA processing. The result was a significant reduction in the level of most EV-associated miRNAs in circulation. Furthermore, these adipocyte-derived miRNAs were shown to regulate gene expression in the liver. AT tissue macrophages were also found to release miRNA-containing EVs that effect systemic metabolism when injected into mice^8^. Soon after, the first studies to demonstrate EV-mediated crosstalk between cells in adipose tissue in vivo were published. The first used genetically labeled adipocyte membranes and tagged endothelial cell caveolin-1 to demonstrate a robust exchange of cellular material between cells within adipose tissue in mice^9^. This was found to be through EVs and was regulated by feeding and fasting. The second study demonstrated that adipocyte EVs are lipid-filled and are taken up by local macrophages in vivo^1^. These EVs functioned as a source of lipid for macrophages but also promoted differentiation of bone marrow stem cells into adipose tissue macrophage-like cells. Together, these studies made a strong case for the existence of an unexplored network of signaling that regulates metabolism within the AT and between the AT and other organs.

## The unique challenges of isolating adipose tissues EVs

**Fig. 1:**
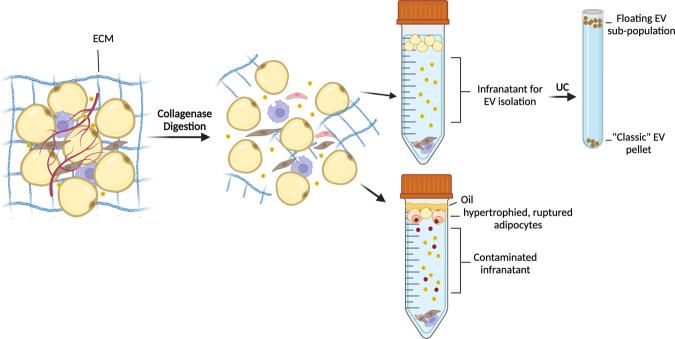
Challenges of EV isolation from adipose tissue. Collagenase digestion is required to liberate cells from the extracellular matrix (ECM) and produce a single-cell suspension. Adipocytes are fragile under these conditions and may burst, particularly if they are hypertrophied. If adipocytes are ruptured, the infranatant, where tissue EVs are found, will be contaminated with intracellular vesicles. Once a clean infranatant is collected, EVs can be isolated through various techniques, however if ultracentrifugation (UC) is used a population of triglyceride-containing EVs may not pellet and, instead, float. Images were created with BioRender.com.

There are important aspects of AT EV research that should be considered when assessing data that demonstrates a physiological effect of EVs. Firstly, adipose tissue has unique biophysical properties that contribute to challenges for clean tissue EV isolation. Adipose tissue is a soft connective tissue that is primarily composed of lipid-filled adipocytes surrounded by a dense but mailable extracellular matrix. Support cells like endothelial cells, fibroblasts and immune cells contribute to this specialized microenvironment that allows for remodeling of the extracellular matrix and rapid expansion or contraction of the tissue depending on the nutrient supply. In the obese state adipocytes become engorged with lipid and prone to apoptosis. Obese adipose tissue is characterized by excessive collagen deposition and inflammation that makes for a rigid and less dynamic tissue. Isolation of EVs from tissues in general requires a collagenase digestion step to liberate the cells and solubilize the interstitial fluid where the EVs are found^10^. The digested sample is centrifuged to pellet stromal cells and float adipocytes so that the interstitial fluid can be harvested (Fig. 1). The key to a pure EV isolation is to ensure the cells remain unbroken during the process so that no intracellular vesicles contaminate the EV preparation. Adipocytes are fragile when separated from the extracellular matrix and so they tend to rupture, particularly if the sample is from obese mice where adipocytes are severely hypertrophied (Fig. 1). Evidence of ruptured adipocytes is oil floating on the top of the sample after centrifugation, which appears distinctly different from the floating, intact adipocytes. Special care must be taken to optimize the digestion conditions to maximize the trade-off between EV yield and purity. This concept is also true for the culturing of primary adipocytes for EV harvesting. Because adipocytes float on the surface of the culture media they are unstable and prone to de-differentiating or rupturing^11^. This should be a serious consideration in studies that use these EVs to demonstrate functional outcomes. A measure of adipocyte viability at the end of the culture period would be a beneficial standard practice. Primary floated adipocyte culture does have value because the in vivo physiological state of the cell may be maintained in culture such as obese or insulin resistance phenotypes. This is unlike the alternative, which is to differentiate adipocytes in vitro from the stromal vascular fraction. In the differentiated culture the adipocytes adhere to the culture plate and tend to be healthy and more resilient, but any in vivo phenotypes would likely be lost. In this scenario, the differentiated adipocytes are a reliable tool for EV collection under treatment conditions. If the in vivo phenotype is the desired readout for the culture experiment, it may be required to implement a ceiling culture or membrane culture to maintain adipocyte integrity^11^. However, one should assess the potential utility of each option by how many EVs are needed for downstream applications.

Many research groups have opted to use whole adipose tissue explant culture for EV production and harvest. Tissue should be cut into small pieces (~2–5 mm^3^) and only cultured short term to avoid necrosis, apoptosis or loss of adipose tissue identity^11^. Because adipocytes maintain their native microenvironment, tissue explants may provide clean EV samples, however, these EVs are derived from all cells in the tissue, not just adipocytes. This should be clearly stated in published works to avoid confusion. It is true that adipocyte EVs may constitute the majority of EVs from adipose tissue explants^1^ but, EVs from other cells in the tissue have robust signaling capacity that would confound interpretation of a functional effect^8^.

The last challenge is of adipose tissue EV isolation is the inefficiency of commonly used techniques to isolate adipocyte EVs. At least a subpopulation of adipocyte-derived EVs contain a substantial amount of neutral lipid and fatty acids, which causes EVs to float to the top of the tube during ultracentrifugation (Fig. 1)^1,12^. For this reason, Flaherty et. al. suggests the use of ultrafiltration and/or size-exclusion chromatography for isolation of EVs from adipocyte cultures or adipose tissue explant cultures. Therefore, when interpreting functional data, it is important to consider the method of EV isolation, as the isolated EV population may only be the subpopulation that pelleted during ultracentrifugation.

## The barriers to studying EVs in physiology

**Fig. 2:**
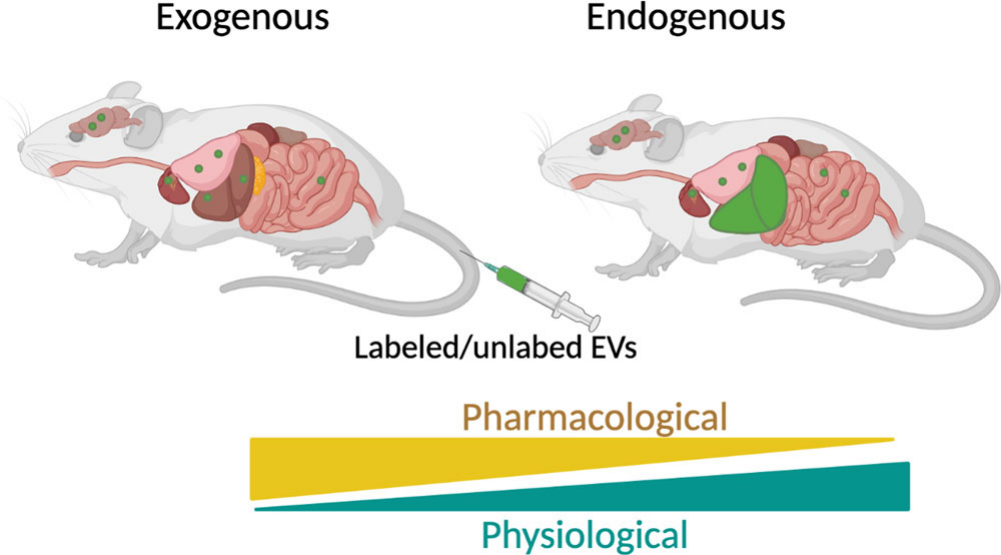
Experimental approaches for studying EV dynamics. EVs labeled with radioactivity, luminescence or florescence are routinely injected to study the kinetics and biodistribution of EVs. Unlabeled EVs are also injected to study the signaling effects on organ. Endogenous EVs can tracked or manipulated using genetic mouse models that enable cell-type specificity. In general, high doses of exogenous EVs provide valuable insight into therapeutic potential, whereas studying endogenous EVs provides the most physiologically relevant information. Images were created with BioRender.com.

Technical issues aside, the principal factor holding the field back from a comprehensive understanding of all EVs in physiology is our lack of in vivo model systems to track and modulate EV production in a cell-type specific way. Ideally this would be done in an inducible manner as to study the kinetics and dynamics of EV production and signaling. Most of the EV functional readouts in the field rely on injection of EVs into mice, or treatment of a cell type with EVs purified from a donor cell type. One cannot argue against the potential artifacts created by exposing cells or organs to large doses of cell-type-specific EVs that may never occur under physiological conditions. Without knowledge of the cell type EV release dynamics, half-life kinetics, preference for retention of EVs in the tissue of the producing cell versus entry into the blood, and cell targeting, it is challenging to estimate the true physiological effect of a cell type-specific EV population. It is important to note that EVs are considered a strong candidate for therapeutic treatment of various diseases or drug delivery^13^. Therefore, injections or treatments of what may be supraphysiological levels of EVs may provide valuable information about the therapeutic potential (Fig. 2).

The biggest hurdle for manipulation of endogenous EV production is finding a target protein or pathway that will do so with minimal disruption to cellular processes unrelated to EVs. For example, knocking out ESCRT proteins effectively suppresses EV production, but these proteins are also involved in various other intracellular trafficking processes in the cell^14^ making it challenging to assign any effect to EVs and not disruption of cell function. Tools that are sensitive enough to track endogenous EVs are also needed. Great strides have been taken to track EVs in zebrafish models which have already offered insights into EV dynamics^15^, however, establishing mammalian models are more challenging. A common approach is to genetically label the plasma membrane or an EV-enriched protein, like tetraspanins, with a fluorescent tag. This approach seems to be effective for tracking of EVs in the tissue^1,9,16^ but is not sensitive enough to track endogenous EV targeting to other organs. Injections of fluorescent, luminescent or radiolabeled EVs are effective for biodistribution studies (Fig. 2), but again, the risk is artifacts produced by injection of supraphysiological levels of EVs.

## Outlook

These challenges are not trivial to solve, but with collaboration and creativity the field will surmount these obstacles. Even now, with the available tools there is exciting and foundational work being done that is leading us closer to understanding how AT EVs contribute to physiological and pathophysiological processes.
